# 2-Chloro-8,8-dimethyl-8,9-dihydro-7*H*-chromeno[2,3-*b*]quinoline-10,12-dione

**DOI:** 10.1107/S1600536813001062

**Published:** 2013-01-19

**Authors:** Thothadri Srinivasan, Panneerselvam Yuvaraj, Boreddy S. R. Reddy, Devadasan Velmurugan

**Affiliations:** a Centre of Advanced Study in Crystallography and Biophysics, University of Madras, Guindy Campus, Chennai 600 025, India; b Industrial Chemistry Laboratory, Central Leather Research Institute, Adyar, Chennai 600 020, India

## Abstract

The asymmetric unit of the title compound, C_18_H_14_ClNO_3_, contains two independent mol­ecules (*A* and *B*). In both mol­ecules, the cyclo­hexa­none ring has a chair conformation. The dihedral angles between the pyran ring and the pyridine and chloro­phenyl rings are 2.13 (9) and 2.19 (9)°, respectively, in *A*, and 0.82 (9) and 1.93 (9)°, respectively, in *B*. The carbonyl O atoms deviate from the pyran and benzene rings to which they are attached by −0.092 (2) and 0.064 (2) Å, respectively, in *A*, and by −0.080 (2) and −0.063 (2) Å, respectively, in *B*. In the crystal, the *A* mol­ecules are linked *via* C—H⋯O hydrogen bonds, forming double-stranded chains along [100]. They lie parallel to the double-stranded chains formed by the *B* mol­ecules, which are also linked *via* C—H⋯O hydrogen bonds. The chains stack up the *c* axis in an –*A*–*A*–*B*–*B*–*A*–*A*– manner, with a number of π–π inter­actions involving *A* and *B* mol­ecules; the centroid–centroid distances vary from 3.4862 (11) to 3.6848 (11) Å

## Related literature

For the uses and biological importance of diketones, see: Bennett *et al.* (1999 ▶); Sato *et al.* (2008 ▶). For a related structure, see: Öztürk Yildirim *et al.* (2012 ▶).
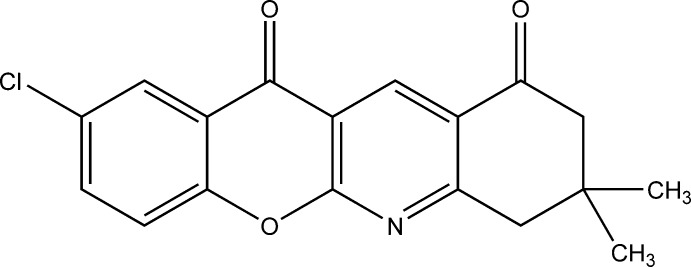


## Experimental

### 

#### Crystal data

C_18_H_14_ClNO_3_
*M* *_r_* = 327.75
Triclinic, 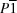

*a* = 11.4135 (4) Å
*b* = 11.6663 (5) Å
*c* = 13.5810 (6) Å
α = 69.265 (3)°
β = 73.868 (2)°
γ = 64.998 (5)°
*V* = 1515.11 (12) Å^3^
*Z* = 4
Mo *K*α radiation
μ = 0.27 mm^−1^
*T* = 293 K
0.30 × 0.25 × 0.20 mm

#### Data collection

Bruker SMART APEXII area-detector diffractometer
Absorption correction: multi-scan (*SADABS*; Bruker, 2008 ▶) *T*_min_ = 0.924, *T*_max_ = 0.949
26747 measured reflections
7488 independent reflections
5384 reflections with *I* > 2σ(*I*)
*R*_int_ = 0.033

#### Refinement

*R*[*F* ^2^ > 2σ(*F* ^2^)] = 0.053
*wR*(*F* ^2^) = 0.167
*S* = 0.99
7488 reflections
419 parameters
H-atom parameters constrained
Δρ_max_ = 0.27 e Å^−3^
Δρ_min_ = −0.33 e Å^−3^

### 

Data collection: *APEX2* (Bruker, 2008 ▶); cell refinement: *SAINT* (Bruker, 2008 ▶); data reduction: *SAINT*; program(s) used to solve structure: *SHELXS97* (Sheldrick, 2008 ▶); program(s) used to refine structure: *SHELXL97* (Sheldrick, 2008 ▶); molecular graphics: *ORTEP-3 for Windows* (Farrugia, 2012 ▶); software used to prepare material for publication: *SHELXL97* and *PLATON* (Spek, 2009 ▶).

## Supplementary Material

Crystal structure: contains datablock(s) global, I. DOI: 10.1107/S1600536813001062/su2550sup1.cif

Structure factors: contains datablock(s) I. DOI: 10.1107/S1600536813001062/su2550Isup2.hkl

Supplementary material file. DOI: 10.1107/S1600536813001062/su2550Isup3.cml

Additional supplementary materials:  crystallographic information; 3D view; checkCIF report

## Figures and Tables

**Table 1:** Hydrogen-bond geometry (Å, °)

*D*—H⋯*A*	*D*—H	H⋯*A*	*D*⋯*A*	*D*—H⋯*A*
C2—H2⋯O3^i^	0.93	2.33	3.255 (3)	174
C2′—H2′⋯O3′^i^	0.93	2.38	3.309 (3)	173

Symmetry code: (i) 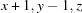.

**Fig. 1.**
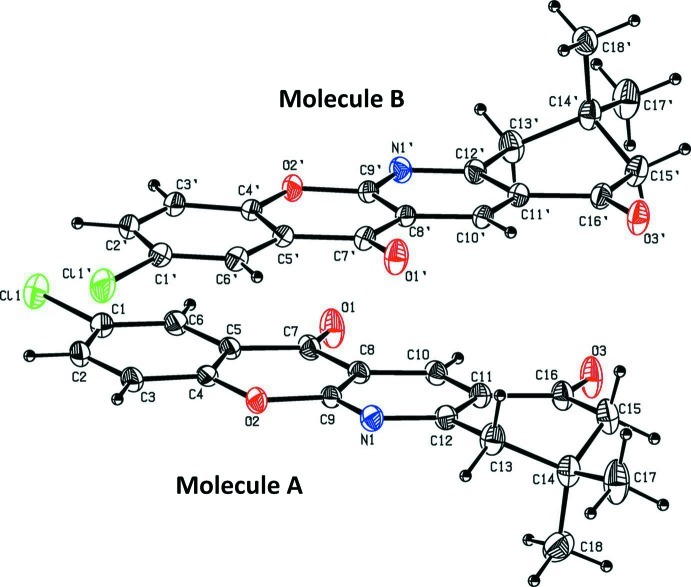
The molecular structure of the two independent molecules (A and B) of the title compound, showing the atom numbering. Displacement ellipsoids are drawn at the 30% probability level.

**Fig. 2.**
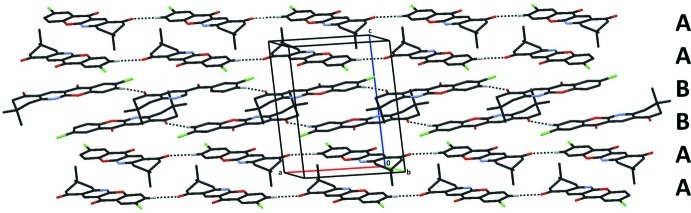
The crystal packing of the title compound viewed along the b axis. H-atoms not involved in hydrogen bonding (dashed lines) have been omitted for clarity.

## supplementary crystallographic information

### Comment

Diketones are popular in organic synthesis for their applications in biology and medicine. They are known to exhibit antioxidants, antitumour and antibacterial activities (Bennett *et al.*, 1999). They are also key intermediates in the preparation of various heterocyclic compounds (Sato *et al.*, 2008). The synthesis and crystal structure of the title diketone is reported on herein.

The asymmetric unit of the title compound contains two independent molecules (A and B), as illustrated in Fig. 1. In both molecules the cyclohexanone ring [(C11-C1) in A and (C11'-C16') in B] has a chair conformation. In molecule A the dihedral angle between the pyran ring (O2/C4-C9) and the pyridine ring (N1/C8-C12) is 2.13 (9)°. The dihedral angle between the pyran ring and the phenyl ring (C11-C16) is 4.11 (10)° and the dihedral angle between the pyran ring and the chlorophenyl ring (C1-C6) is 2.19 (9)°. In molecule B the dihedral angle between the pyran ring (O2'/C4'-C9') and the pyridine ring (N1'/C8'-C12') is 0.82 (9)°. The dihedral angle between the pyran ring and the phenyl ring (C11'-C16') is 8.21 (10)° and the dihedral angle between the pyran ring and the chlorophenyl ring (C1'-C6') is 1.93 (9)°.

In molecule A the carbonyl oxygen atoms O1 and O3 attached to the pyran ring (O2/C4-C9) and the phenyl ring (C11-C16) deviate by -0.0915 (22)Å and 0.0643 (24)Å, respectively. In molecule B the carbonyl oxygen atoms O1' and O3' attached to the pyran ring (O2'/C4'-C9') and the phenyl ring (C11'-C16') deviate by -0.096 (2) Å and -0.063 (2) Å, respectively.

In A the pyridine ring makes a dihedral angle of 6.23 (10)° with the phenyl ring and a dihedral angle of 4.31 (10)° with the chlorophenyl ring. In B the pyridine ring makes a dihedral angle of 8.85 (10)° with the phenyl ring and a dihedral angle of 2.42 (10)° with the chloro phenyl ring. The chlorine atoms Cl1 and Cl1' deviated by -0.0423 (8) Å and 0.0598 (8) Å, respectively, from the phenyl ring to which they are attached.

In the crystal, the A molecules are linked via C-H···O hydrogen bonds to form chains along the a axis. They lie parallel to the chains formed by the B molecules which are also linked via C-H···O hydrogen bonds (Table 1 and Fig. 2). The A and B molecules are also linked via a number of π-π interactions; these include Cg1···Cg3^i^ 3.4969 (11) Å; Cg1···Cg11 3.4862 (11) Å; Cg2···Cg11 3.6727 (11) Å; Cg2···Cg12 3.6848 (11) Å; Cg11···Cg13^i^ 3.5101 (11) Å; symmetry code: (i) -x+1, -y+1, -z; where Cg1, Cg2, Cg3, Cg11, Cg12, Cg13 are the centroids of rings O2/C4-C9, N1/C8-C12, C1-C6, O2'/C4'-C9', N1'/C9'-C12' and C1'-C6', respectively.

### Experimental

2-Amino-4-oxo-4H-chromene-3-carbaldehyde ( 100 mg, 1 mmol) was reacted with 5,5-dimethylcyclohexane-1,3-dione( 88 mg, 1.2 mmol) in the presence of yeterbium triflate [Yb(oft)_3_] (98 mg, 0.3 mmol) after stirring. All the reactants were dissolved in xylene (5 ml). The reaction mixture was refluxed at 398 K for 12 hours. The reaction mixture was extracted with ethyl acetate/Hexane 40:60 (v/v). The completion of the reaction was monitored by TLC. The final product was purified by column chromatography (ethyl acetate/hexane) giving the pure title compound [Yield = 80%]. Single crystals suitable for X-ray diffraction were obtained by slow evaporation of a solution of the title compound in ethyl acetate at room temperature.

### Refinement

The hydrogen atoms were placed in calculated positions and treated as riding atoms: C—H = 0.93 Å to 0.97 Å, with *U*_iso_(H) = 1.5*U*_eq_(C) for methyl H atoms and = 1.2*U*_eq_(C) for other H atoms.

### Crystal data

**Table:**

C_18_H_14_ClNO_3_	*Z* = 4
*M**_r_* = 327.75	*F*(000) = 680
Triclinic, *P*1	*D*_x_ = 1.437 Mg m^−^^3^
Hall symbol: -P 1	Mo *K*α radiation, λ = 0.71073 Å
*a* = 11.4135 (4) Å	Cell parameters from 7488 reflections
*b* = 11.6663 (5) Å	θ = 1.6–28.4°
*c* = 13.5810 (6) Å	µ = 0.27 mm^−^^1^
α = 69.265 (3)°	*T* = 293 K
β = 73.868 (2)°	Block, colourless
γ = 64.998 (5)°	0.30 × 0.25 × 0.20 mm
*V* = 1515.11 (12) Å^3^	

### Data collection

**Table:**

Bruker SMART APEXII area-detector diffractometer	7488 independent reflections
Radiation source: fine-focus sealed tube	5384 reflections with *I* > 2σ(*I*)
Graphite monochromator	*R*_int_ = 0.033
ω and φ scans	θ_max_ = 28.4°, θ_min_ = 1.6°
Absorption correction: multi-scan (*SADABS*; Bruker, 2008)	*h* = −15→14
*T*_min_ = 0.924, *T*_max_ = 0.949	*k* = −15→15
26747 measured reflections	*l* = −18→18

### Refinement

**Table:**

Refinement on *F*^2^	Primary atom site location: structure-invariant direct methods
Least-squares matrix: full	Secondary atom site location: difference Fourier map
*R*[*F*^2^ > 2σ(*F*^2^)] = 0.053	Hydrogen site location: inferred from neighbouring sites
*wR*(*F*^2^) = 0.167	H-atom parameters constrained
*S* = 0.99	*w* = 1/[σ^2^(*F*_o_^2^) + (0.0783*P*)^2^ + 0.7873*P*] where *P* = (*F*_o_^2^ + 2*F*_c_^2^)/3
7488 reflections	(Δ/σ)_max_ < 0.001
419 parameters	Δρ_max_ = 0.27 e Å^−^^3^
0 restraints	Δρ_min_ = −0.33 e Å^−^^3^

### Special details

**Table:**

Geometry. All esds (except the esd in the dihedral angle between two l.s. planes) are estimated using the full covariance matrix. The cell esds are taken into account individually in the estimation of esds in distances, angles and torsion angles; correlations between esds in cell parameters are only used when they are defined by crystal symmetry. An approximate (isotropic) treatment of cell esds is used for estimating esds involving l.s. planes.
Refinement. Refinement of F^2^ against ALL reflections. The weighted R-factor wR and goodness of fit S are based on F^2^, conventional R-factors R are based on F, with F set to zero for negative F^2^. The threshold expression of F^2^ > 2sigma(F^2^) is used only for calculating R-factors(gt) etc. and is not relevant to the choice of reflections for refinement. R-factors based on F^2^ are statistically about twice as large as those based on F, and R- factors based on ALL data will be even larger.

### Fractional atomic coordinates and isotropic or equivalent isotropic displacement parameters (Å^2^)

**Table:**

	*x*	*y*	*z*	*U*_iso_*/*U*_eq_	
C1	0.5452 (2)	0.2391 (2)	0.17141 (17)	0.0507 (5)	
C1'	0.7356 (2)	0.4947 (2)	0.32726 (17)	0.0547 (5)	
C2	0.6544 (2)	0.2742 (2)	0.14200 (17)	0.0528 (5)	
H2	0.7376	0.2098	0.1389	0.063*	
C2'	0.7043 (2)	0.3837 (2)	0.35361 (17)	0.0558 (6)	
H2'	0.7703	0.3019	0.3552	0.067*	
C3'	0.5750 (2)	0.3948 (2)	0.37743 (17)	0.0525 (5)	
H3'	0.5532	0.3209	0.3951	0.063*	
C3	0.64025 (19)	0.4033 (2)	0.11754 (17)	0.0492 (5)	
H3	0.7135	0.4271	0.0973	0.059*	
C4	0.51544 (18)	0.4985 (2)	0.12322 (14)	0.0411 (4)	
C4'	0.4777 (2)	0.51767 (19)	0.37477 (15)	0.0432 (4)	
C5	0.40526 (18)	0.4646 (2)	0.15103 (15)	0.0422 (4)	
C5'	0.5084 (2)	0.62854 (19)	0.35132 (15)	0.0435 (4)	
C6'	0.6394 (2)	0.6162 (2)	0.32709 (17)	0.0511 (5)	
H6'	0.6617	0.6895	0.3109	0.061*	
C6	0.4216 (2)	0.3327 (2)	0.17485 (17)	0.0489 (5)	
H6	0.3490	0.3083	0.1930	0.059*	
C7'	0.4033 (2)	0.75624 (19)	0.35395 (16)	0.0448 (4)	
C7	0.27378 (19)	0.5668 (2)	0.15261 (17)	0.0458 (5)	
C8	0.27349 (17)	0.6992 (2)	0.13091 (15)	0.0401 (4)	
C8'	0.27101 (19)	0.75360 (18)	0.37550 (14)	0.0386 (4)	
C9	0.38973 (17)	0.7232 (2)	0.10728 (14)	0.0395 (4)	
C9'	0.25063 (19)	0.63696 (18)	0.39621 (15)	0.0403 (4)	
C10	0.15855 (18)	0.8078 (2)	0.13264 (16)	0.0431 (4)	
H10	0.0782	0.7981	0.1466	0.052*	
C10'	0.15975 (19)	0.86517 (18)	0.37749 (15)	0.0406 (4)	
H10'	0.1675	0.9457	0.3645	0.049*	
C11	0.16353 (18)	0.9292 (2)	0.11373 (16)	0.0433 (4)	
C11'	0.0377 (2)	0.85759 (19)	0.39857 (16)	0.0419 (4)	
C12	0.28618 (19)	0.9417 (2)	0.09298 (16)	0.0452 (4)	
C12'	0.0290 (2)	0.73483 (19)	0.41838 (16)	0.0443 (4)	
C13	0.2948 (2)	1.0714 (2)	0.0769 (2)	0.0595 (6)	
H13A	0.3744	1.0758	0.0288	0.071*	
H13B	0.3009	1.0774	0.1446	0.071*	
C13'	−0.1015 (2)	0.7213 (2)	0.4426 (2)	0.0604 (6)	
H13C	−0.0945	0.6345	0.4897	0.072*	
H13D	−0.1256	0.7286	0.3770	0.072*	
C14	0.1788 (2)	1.1890 (2)	0.0318 (2)	0.0574 (6)	
C14'	−0.2096 (2)	0.8249 (2)	0.4948 (2)	0.0547 (5)	
C15'	−0.2116 (2)	0.9614 (2)	0.4250 (2)	0.0583 (6)	
H15A	−0.2399	0.9786	0.3584	0.070*	
H15B	−0.2753	1.0267	0.4606	0.070*	
C15	0.0525 (2)	1.1739 (2)	0.1015 (2)	0.0668 (7)	
H15C	0.0477	1.1832	0.1709	0.080*	
H15D	−0.0214	1.2446	0.0697	0.080*	
C16	0.0408 (2)	1.0454 (2)	0.1164 (2)	0.0532 (5)	
C16'	−0.0816 (2)	0.97710 (19)	0.40067 (17)	0.0470 (5)	
C17	0.1858 (3)	1.3168 (3)	0.0318 (3)	0.0908 (11)	
H17A	0.2678	1.3235	−0.0077	0.136*	
H17B	0.1789	1.3172	0.1038	0.136*	
H17C	0.1153	1.3900	−0.0007	0.136*	
C17'	−0.3419 (3)	0.8143 (3)	0.5042 (3)	0.0808 (9)	
H17D	−0.4098	0.8793	0.5368	0.121*	
H17E	−0.3400	0.7283	0.5472	0.121*	
H17F	−0.3586	0.8286	0.4345	0.121*	
C18	0.1796 (3)	1.1930 (3)	−0.0825 (2)	0.0802 (8)	
H18A	0.2588	1.2024	−0.1261	0.120*	
H18B	0.1058	1.2663	−0.1102	0.120*	
H18C	0.1746	1.1130	−0.0826	0.120*	
C18'	−0.1844 (2)	0.8035 (3)	0.6060 (2)	0.0637 (6)	
H18D	−0.2542	0.8674	0.6386	0.096*	
H18E	−0.1031	0.8129	0.6004	0.096*	
H18F	−0.1803	0.7168	0.6487	0.096*	
N1	0.39826 (15)	0.83855 (18)	0.08958 (14)	0.0470 (4)	
N1'	0.13495 (18)	0.62525 (16)	0.41741 (14)	0.0474 (4)	
O1	0.17423 (15)	0.54410 (18)	0.16973 (18)	0.0723 (5)	
O1'	0.42395 (16)	0.85595 (16)	0.33981 (16)	0.0663 (5)	
O2'	0.35174 (14)	0.52027 (13)	0.39693 (12)	0.0485 (3)	
O2	0.50909 (12)	0.62565 (14)	0.10098 (11)	0.0463 (3)	
O3	−0.06431 (15)	1.03402 (18)	0.13335 (19)	0.0777 (6)	
O3'	−0.07287 (16)	1.08344 (15)	0.38172 (16)	0.0648 (5)	
Cl1'	0.89836 (6)	0.48087 (7)	0.29361 (6)	0.0808 (2)	
Cl1	0.56258 (7)	0.07546 (6)	0.20440 (6)	0.0733 (2)	

### Atomic displacement parameters (Å^2^)

**Table:**

	*U*^11^	*U*^22^	*U*^33^	*U*^12^	*U*^13^	*U*^23^
C1	0.0506 (12)	0.0466 (11)	0.0461 (11)	−0.0068 (9)	−0.0091 (9)	−0.0145 (9)
C1'	0.0457 (11)	0.0560 (13)	0.0444 (11)	−0.0033 (10)	−0.0101 (9)	−0.0091 (9)
C2	0.0416 (11)	0.0522 (12)	0.0490 (12)	0.0025 (9)	−0.0083 (9)	−0.0185 (10)
C2'	0.0551 (13)	0.0442 (11)	0.0447 (11)	0.0062 (10)	−0.0117 (9)	−0.0120 (9)
C3'	0.0608 (13)	0.0367 (10)	0.0450 (11)	−0.0027 (9)	−0.0093 (9)	−0.0112 (8)
C3	0.0332 (9)	0.0575 (13)	0.0469 (11)	−0.0066 (9)	−0.0038 (8)	−0.0166 (9)
C4	0.0350 (9)	0.0468 (11)	0.0336 (9)	−0.0076 (8)	−0.0048 (7)	−0.0110 (8)
C4'	0.0483 (11)	0.0365 (10)	0.0341 (9)	−0.0051 (8)	−0.0065 (8)	−0.0097 (7)
C5	0.0338 (9)	0.0493 (11)	0.0369 (9)	−0.0075 (8)	−0.0043 (7)	−0.0141 (8)
C5'	0.0464 (11)	0.0370 (10)	0.0361 (9)	−0.0053 (8)	−0.0077 (8)	−0.0083 (7)
C6'	0.0470 (11)	0.0468 (11)	0.0470 (11)	−0.0082 (9)	−0.0095 (9)	−0.0073 (9)
C6	0.0449 (11)	0.0517 (12)	0.0473 (11)	−0.0138 (9)	−0.0056 (9)	−0.0157 (9)
C7'	0.0469 (11)	0.0342 (10)	0.0457 (11)	−0.0085 (8)	−0.0088 (8)	−0.0082 (8)
C7	0.0339 (9)	0.0511 (11)	0.0497 (11)	−0.0112 (8)	−0.0024 (8)	−0.0184 (9)
C8	0.0304 (8)	0.0490 (11)	0.0378 (9)	−0.0102 (8)	−0.0050 (7)	−0.0136 (8)
C8'	0.0450 (10)	0.0310 (9)	0.0356 (9)	−0.0095 (8)	−0.0058 (7)	−0.0097 (7)
C9	0.0274 (8)	0.0468 (10)	0.0374 (9)	−0.0075 (7)	−0.0050 (7)	−0.0107 (8)
C9'	0.0474 (10)	0.0304 (9)	0.0375 (9)	−0.0086 (8)	−0.0046 (8)	−0.0111 (7)
C10	0.0287 (8)	0.0523 (11)	0.0471 (11)	−0.0125 (8)	−0.0043 (7)	−0.0156 (9)
C10'	0.0469 (10)	0.0277 (8)	0.0429 (10)	−0.0105 (8)	−0.0068 (8)	−0.0082 (7)
C11	0.0306 (9)	0.0465 (11)	0.0479 (11)	−0.0087 (8)	−0.0063 (7)	−0.0131 (9)
C11'	0.0445 (10)	0.0338 (9)	0.0436 (10)	−0.0110 (8)	−0.0059 (8)	−0.0106 (8)
C12	0.0341 (9)	0.0500 (11)	0.0473 (11)	−0.0127 (8)	−0.0071 (8)	−0.0108 (9)
C12'	0.0477 (11)	0.0365 (10)	0.0477 (11)	−0.0134 (8)	−0.0038 (8)	−0.0150 (8)
C13	0.0414 (11)	0.0538 (13)	0.0837 (17)	−0.0162 (10)	−0.0119 (11)	−0.0185 (12)
C13'	0.0535 (13)	0.0485 (12)	0.0853 (17)	−0.0205 (10)	−0.0039 (12)	−0.0276 (12)
C14	0.0433 (11)	0.0438 (11)	0.0814 (16)	−0.0128 (9)	−0.0082 (11)	−0.0174 (11)
C14'	0.0394 (10)	0.0427 (11)	0.0792 (16)	−0.0130 (9)	−0.0093 (10)	−0.0153 (11)
C15'	0.0441 (11)	0.0435 (11)	0.0791 (16)	−0.0095 (9)	−0.0169 (11)	−0.0091 (11)
C15	0.0426 (12)	0.0531 (14)	0.100 (2)	−0.0092 (10)	0.0002 (12)	−0.0335 (13)
C16	0.0349 (10)	0.0497 (12)	0.0690 (14)	−0.0089 (9)	−0.0048 (9)	−0.0194 (10)
C16'	0.0458 (11)	0.0342 (10)	0.0533 (12)	−0.0083 (8)	−0.0090 (9)	−0.0096 (8)
C17	0.0578 (16)	0.0537 (15)	0.164 (3)	−0.0169 (13)	−0.0138 (18)	−0.0396 (18)
C17'	0.0481 (14)	0.0622 (16)	0.133 (3)	−0.0216 (12)	−0.0126 (15)	−0.0261 (17)
C18	0.085 (2)	0.0621 (16)	0.0777 (19)	−0.0252 (15)	−0.0165 (15)	0.0008 (14)
C18'	0.0492 (12)	0.0575 (14)	0.0698 (16)	−0.0152 (11)	0.0025 (11)	−0.0143 (12)
N1	0.0304 (8)	0.0511 (10)	0.0536 (10)	−0.0118 (7)	−0.0061 (7)	−0.0111 (8)
N1'	0.0508 (10)	0.0352 (8)	0.0536 (10)	−0.0139 (7)	−0.0004 (8)	−0.0170 (7)
O1	0.0344 (8)	0.0593 (10)	0.1242 (16)	−0.0154 (7)	−0.0048 (9)	−0.0333 (10)
O1'	0.0514 (9)	0.0411 (8)	0.1038 (14)	−0.0149 (7)	−0.0113 (9)	−0.0193 (8)
O2'	0.0505 (8)	0.0299 (7)	0.0568 (9)	−0.0071 (6)	−0.0044 (6)	−0.0141 (6)
O2	0.0271 (6)	0.0479 (8)	0.0540 (8)	−0.0069 (6)	−0.0029 (5)	−0.0133 (6)
O3	0.0295 (8)	0.0578 (10)	0.1401 (18)	−0.0059 (7)	−0.0090 (9)	−0.0355 (11)
O3'	0.0524 (9)	0.0339 (8)	0.0981 (13)	−0.0090 (7)	−0.0087 (9)	−0.0167 (8)
Cl1'	0.0443 (3)	0.0753 (5)	0.0916 (5)	0.0002 (3)	−0.0115 (3)	−0.0129 (4)
Cl1	0.0716 (4)	0.0471 (3)	0.0927 (5)	−0.0067 (3)	−0.0227 (3)	−0.0194 (3)

### Geometric parameters (Å, º)

**Table:**

C1—C6	1.372 (3)	C11—C12	1.409 (3)
C1—C2	1.385 (3)	C11—C16	1.484 (3)
C1—Cl1	1.735 (2)	C11'—C12'	1.402 (3)
C1'—C6'	1.377 (3)	C11'—C16'	1.483 (3)
C1'—C2'	1.388 (4)	C12—N1	1.339 (3)
C1'—Cl1'	1.736 (2)	C12—C13	1.492 (3)
C2—C3	1.370 (3)	C12'—N1'	1.339 (3)
C2—H2	0.9300	C12'—C13'	1.497 (3)
C2'—C3'	1.379 (3)	C13—C14	1.527 (3)
C2'—H2'	0.9300	C13—H13A	0.9700
C3'—C4'	1.389 (3)	C13—H13B	0.9700
C3'—H3'	0.9300	C13'—C14'	1.532 (3)
C3—C4	1.390 (3)	C13'—H13C	0.9700
C3—H3	0.9300	C13'—H13D	0.9700
C4—O2	1.378 (2)	C14—C17	1.527 (3)
C4—C5	1.390 (3)	C14—C18	1.533 (4)
C4'—O2'	1.374 (3)	C14—C15	1.531 (3)
C4'—C5'	1.388 (3)	C14'—C18'	1.529 (4)
C5—C6	1.395 (3)	C14'—C17'	1.533 (3)
C5—C7	1.470 (3)	C14'—C15'	1.533 (3)
C5'—C6'	1.393 (3)	C15'—C16'	1.503 (3)
C5'—C7'	1.471 (3)	C15'—H15A	0.9700
C6'—H6'	0.9300	C15'—H15B	0.9700
C6—H6	0.9300	C15—C16	1.499 (3)
C7'—O1'	1.222 (2)	C15—H15C	0.9700
C7'—C8'	1.468 (3)	C15—H15D	0.9700
C7—O1	1.216 (2)	C16—O3	1.212 (3)
C7—C8	1.466 (3)	C16'—O3'	1.216 (2)
C8—C10	1.387 (3)	C17—H17A	0.9600
C8—C9	1.398 (3)	C17—H17B	0.9600
C8'—C10'	1.384 (3)	C17—H17C	0.9600
C8'—C9'	1.395 (3)	C17'—H17D	0.9600
C9—N1	1.322 (3)	C17'—H17E	0.9600
C9—O2	1.361 (2)	C17'—H17F	0.9600
C9'—N1'	1.326 (3)	C18—H18A	0.9600
C9'—O2'	1.362 (2)	C18—H18B	0.9600
C10—C11	1.371 (3)	C18—H18C	0.9600
C10—H10	0.9300	C18'—H18D	0.9600
C10'—C11'	1.377 (3)	C18'—H18E	0.9600
C10'—H10'	0.9300	C18'—H18F	0.9600
			
C6—C1—C2	120.8 (2)	C11'—C12'—C13'	120.59 (18)
C6—C1—Cl1	118.75 (18)	C12—C13—C14	113.78 (19)
C2—C1—Cl1	120.41 (17)	C12—C13—H13A	108.8
C6'—C1'—C2'	121.0 (2)	C14—C13—H13A	108.8
C6'—C1'—Cl1'	119.3 (2)	C12—C13—H13B	108.8
C2'—C1'—Cl1'	119.64 (17)	C14—C13—H13B	108.8
C3—C2—C1	120.21 (19)	H13A—C13—H13B	107.7
C3—C2—H2	119.9	C12'—C13'—C14'	113.01 (18)
C1—C2—H2	119.9	C12'—C13'—H13C	109.0
C3'—C2'—C1'	119.8 (2)	C14'—C13'—H13C	109.0
C3'—C2'—H2'	120.1	C12'—C13'—H13D	109.0
C1'—C2'—H2'	120.1	C14'—C13'—H13D	109.0
C2'—C3'—C4'	119.2 (2)	H13C—C13'—H13D	107.8
C2'—C3'—H3'	120.4	C13—C14—C17	110.4 (2)
C4'—C3'—H3'	120.4	C13—C14—C18	109.7 (2)
C2—C3—C4	119.4 (2)	C17—C14—C18	109.3 (2)
C2—C3—H3	120.3	C13—C14—C15	108.6 (2)
C4—C3—H3	120.3	C17—C14—C15	109.3 (2)
O2—C4—C3	116.00 (18)	C18—C14—C15	109.5 (2)
O2—C4—C5	123.13 (17)	C18'—C14'—C17'	109.1 (2)
C3—C4—C5	120.9 (2)	C18'—C14'—C13'	110.3 (2)
O2'—C4'—C5'	123.44 (17)	C17'—C14'—C13'	109.4 (2)
O2'—C4'—C3'	115.37 (19)	C18'—C14'—C15'	109.7 (2)
C5'—C4'—C3'	121.2 (2)	C17'—C14'—C15'	109.6 (2)
C4—C5—C6	118.95 (18)	C13'—C14'—C15'	108.7 (2)
C4—C5—C7	120.38 (19)	C16'—C15'—C14'	113.85 (18)
C6—C5—C7	120.65 (18)	C16'—C15'—H15A	108.8
C4'—C5'—C6'	119.05 (18)	C14'—C15'—H15A	108.8
C4'—C5'—C7'	119.92 (19)	C16'—C15'—H15B	108.8
C6'—C5'—C7'	121.02 (19)	C14'—C15'—H15B	108.8
C1'—C6'—C5'	119.6 (2)	H15A—C15'—H15B	107.7
C1'—C6'—H6'	120.2	C16—C15—C14	114.19 (19)
C5'—C6'—H6'	120.2	C16—C15—H15C	108.7
C1—C6—C5	119.7 (2)	C14—C15—H15C	108.7
C1—C6—H6	120.1	C16—C15—H15D	108.7
C5—C6—H6	120.1	C14—C15—H15D	108.7
O1'—C7'—C8'	122.76 (18)	H15C—C15—H15D	107.6
O1'—C7'—C5'	123.1 (2)	O3—C16—C11	120.3 (2)
C8'—C7'—C5'	114.11 (17)	O3—C16—C15	122.1 (2)
O1—C7—C8	122.87 (19)	C11—C16—C15	117.52 (18)
O1—C7—C5	123.4 (2)	O3'—C16'—C11'	120.49 (19)
C8—C7—C5	113.76 (17)	O3'—C16'—C15'	121.99 (19)
C10—C8—C9	116.18 (18)	C11'—C16'—C15'	117.51 (18)
C10—C8—C7	122.19 (17)	C14—C17—H17A	109.5
C9—C8—C7	121.63 (17)	C14—C17—H17B	109.5
C10'—C8'—C9'	116.13 (18)	H17A—C17—H17B	109.5
C10'—C8'—C7'	122.48 (17)	C14—C17—H17C	109.5
C9'—C8'—C7'	121.38 (17)	H17A—C17—H17C	109.5
N1—C9—O2	112.46 (16)	H17B—C17—H17C	109.5
N1—C9—C8	125.55 (17)	C14'—C17'—H17D	109.5
O2—C9—C8	121.99 (18)	C14'—C17'—H17E	109.5
N1'—C9'—O2'	112.55 (16)	H17D—C17'—H17E	109.5
N1'—C9'—C8'	125.45 (17)	C14'—C17'—H17F	109.5
O2'—C9'—C8'	121.99 (18)	H17D—C17'—H17F	109.5
C11—C10—C8	119.92 (17)	H17E—C17'—H17F	109.5
C11—C10—H10	120.0	C14—C18—H18A	109.5
C8—C10—H10	120.0	C14—C18—H18B	109.5
C11'—C10'—C8'	120.33 (17)	H18A—C18—H18B	109.5
C11'—C10'—H10'	119.8	C14—C18—H18C	109.5
C8'—C10'—H10'	119.8	H18A—C18—H18C	109.5
C10—C11—C12	119.19 (18)	H18B—C18—H18C	109.5
C10—C11—C16	120.10 (17)	C14'—C18'—H18D	109.5
C12—C11—C16	120.71 (19)	C14'—C18'—H18E	109.5
C10'—C11'—C12'	118.57 (18)	H18D—C18'—H18E	109.5
C10'—C11'—C16'	120.37 (17)	C14'—C18'—H18F	109.5
C12'—C11'—C16'	121.06 (18)	H18D—C18'—H18F	109.5
N1—C12—C11	121.82 (19)	H18E—C18'—H18F	109.5
N1—C12—C13	117.67 (18)	C9—N1—C12	117.33 (16)
C11—C12—C13	120.49 (18)	C9'—N1'—C12'	117.15 (17)
N1'—C12'—C11'	122.37 (19)	C9'—O2'—C4'	119.03 (15)
N1'—C12'—C13'	117.04 (18)	C9—O2—C4	118.93 (15)
			
C6—C1—C2—C3	1.1 (3)	C8—C10—C11—C16	−179.46 (19)
Cl1—C1—C2—C3	−179.03 (17)	C8'—C10'—C11'—C12'	0.3 (3)
C6'—C1'—C2'—C3'	1.7 (3)	C8'—C10'—C11'—C16'	−179.97 (18)
Cl1'—C1'—C2'—C3'	−178.16 (17)	C10—C11—C12—N1	1.0 (3)
C1'—C2'—C3'—C4'	−0.1 (3)	C16—C11—C12—N1	−179.6 (2)
C1—C2—C3—C4	0.4 (3)	C10—C11—C12—C13	−177.3 (2)
C2—C3—C4—O2	177.88 (18)	C16—C11—C12—C13	2.0 (3)
C2—C3—C4—C5	−1.4 (3)	C10'—C11'—C12'—N1'	−0.1 (3)
C2'—C3'—C4'—O2'	178.43 (18)	C16'—C11'—C12'—N1'	−179.75 (19)
C2'—C3'—C4'—C5'	−1.7 (3)	C10'—C11'—C12'—C13'	179.5 (2)
O2—C4—C5—C6	−178.33 (17)	C16'—C11'—C12'—C13'	−0.2 (3)
C3—C4—C5—C6	0.9 (3)	N1—C12—C13—C14	153.4 (2)
O2—C4—C5—C7	3.2 (3)	C11—C12—C13—C14	−28.2 (3)
C3—C4—C5—C7	−177.54 (18)	N1'—C12'—C13'—C14'	151.8 (2)
O2'—C4'—C5'—C6'	−178.27 (18)	C11'—C12'—C13'—C14'	−27.8 (3)
C3'—C4'—C5'—C6'	1.9 (3)	C12—C13—C14—C17	172.4 (2)
O2'—C4'—C5'—C7'	2.7 (3)	C12—C13—C14—C18	−67.0 (3)
C3'—C4'—C5'—C7'	−177.18 (19)	C12—C13—C14—C15	52.6 (3)
C2'—C1'—C6'—C5'	−1.5 (3)	C12'—C13'—C14'—C18'	−66.8 (3)
Cl1'—C1'—C6'—C5'	178.33 (16)	C12'—C13'—C14'—C17'	173.1 (2)
C4'—C5'—C6'—C1'	−0.3 (3)	C12'—C13'—C14'—C15'	53.5 (3)
C7'—C5'—C6'—C1'	178.77 (19)	C18'—C14'—C15'—C16'	66.6 (3)
C2—C1—C6—C5	−1.6 (3)	C17'—C14'—C15'—C16'	−173.6 (2)
Cl1—C1—C6—C5	178.52 (16)	C13'—C14'—C15'—C16'	−54.1 (3)
C4—C5—C6—C1	0.6 (3)	C13—C14—C15—C16	−53.8 (3)
C7—C5—C6—C1	179.05 (19)	C17—C14—C15—C16	−174.3 (3)
C4'—C5'—C7'—O1'	175.9 (2)	C18—C14—C15—C16	65.9 (3)
C6'—C5'—C7'—O1'	−3.1 (3)	C10—C11—C16—O3	−1.4 (4)
C4'—C5'—C7'—C8'	−3.9 (3)	C12—C11—C16—O3	179.3 (2)
C6'—C5'—C7'—C8'	177.10 (18)	C10—C11—C16—C15	176.5 (2)
C4—C5—C7—O1	175.2 (2)	C12—C11—C16—C15	−2.8 (3)
C6—C5—C7—O1	−3.2 (3)	C14—C15—C16—O3	−152.3 (3)
C4—C5—C7—C8	−4.4 (3)	C14—C15—C16—C11	29.9 (3)
C6—C5—C7—C8	177.16 (18)	C10'—C11'—C16'—O3'	1.5 (3)
O1—C7—C8—C10	2.6 (3)	C12'—C11'—C16'—O3'	−178.8 (2)
C5—C7—C8—C10	−177.72 (18)	C10'—C11'—C16'—C15'	−179.6 (2)
O1—C7—C8—C9	−177.4 (2)	C12'—C11'—C16'—C15'	0.1 (3)
C5—C7—C8—C9	2.2 (3)	C14'—C15'—C16'—O3'	−152.9 (2)
O1'—C7'—C8'—C10'	2.4 (3)	C14'—C15'—C16'—C11'	28.2 (3)
C5'—C7'—C8'—C10'	−177.78 (17)	O2—C9—N1—C12	179.43 (17)
O1'—C7'—C8'—C9'	−177.0 (2)	C8—C9—N1—C12	−1.1 (3)
C5'—C7'—C8'—C9'	2.8 (3)	C11—C12—N1—C9	−0.5 (3)
C10—C8—C9—N1	1.9 (3)	C13—C12—N1—C9	177.90 (19)
C7—C8—C9—N1	−178.08 (19)	O2'—C9'—N1'—C12'	−179.88 (17)
C10—C8—C9—O2	−178.65 (17)	C8'—C9'—N1'—C12'	0.4 (3)
C7—C8—C9—O2	1.4 (3)	C11'—C12'—N1'—C9'	−0.3 (3)
C10'—C8'—C9'—N1'	−0.1 (3)	C13'—C12'—N1'—C9'	−179.85 (19)
C7'—C8'—C9'—N1'	179.33 (18)	N1'—C9'—O2'—C4'	179.10 (16)
C10'—C8'—C9'—O2'	−179.83 (17)	C8'—C9'—O2'—C4'	−1.2 (3)
C7'—C8'—C9'—O2'	−0.4 (3)	C5'—C4'—O2'—C9'	0.0 (3)
C9—C8—C10—C11	−1.2 (3)	C3'—C4'—O2'—C9'	179.84 (17)
C7—C8—C10—C11	178.79 (19)	N1—C9—O2—C4	176.59 (16)
C9'—C8'—C10'—C11'	−0.3 (3)	C8—C9—O2—C4	−2.9 (3)
C7'—C8'—C10'—C11'	−179.69 (18)	C3—C4—O2—C9	−178.66 (17)
C8—C10—C11—C12	−0.2 (3)	C5—C4—O2—C9	0.6 (3)

### Hydrogen-bond geometry (Å, º)

**Table:**

*D*—H···*A*	*D*—H	H···*A*	*D*···*A*	*D*—H···*A*
C2—H2···O3^i^	0.93	2.33	3.255 (3)	174
C2′—H2′···O3′^i^	0.93	2.38	3.309 (3)	173

Symmetry code: (i) *x*+1, *y*−1, *z*.
